# Changes in skin properties are consequence but not cause of green spot in 'WA 38' apples

**DOI:** 10.1371/journal.pone.0356529

**Published:** 2026-08-19

**Authors:** Moritz Knoche, Bruno Carra, Sara Serra, Bishnu P. Khanal, Grecia Hurtado, Simon Sitzenstock, Stefano Musacchi

**Affiliations:** 1 Institute for Horticultural Production Systems, Leibniz-University Hannover, Hannover, Germany; 2 Department of Horticulture, Washington State University, Pullman, Washington, United States of America; 3 Instituto Nacional de Investigación Agropecuaria (INIA), Estación Experimental INIA Las Brujas, Canelones, Uruguay; 4 Tree Fruit Research and Extension Center, Washington State University, Wenatchee, Washington, United States of America; 5 The Pennsylvania State University, Department of Plant Science, State College, Pennsylvania, United States of America; 6 Fruit Research and Extension Center, The Pennsylvania State University, Biglerville, Pennsylvania, United States of America; North-Caucasus Federal University - Pyatigorsk Campus: Severo-Kavkazskij federal'nyj universitet Patigorskij institut filial, RUSSIAN FEDERATION

## Abstract

The green spot (GS) disorder impairs appearance and restricts ‘WA 38’ marketing. The objectives of the present study were to characterize GS in developing ‘WA 38’ fruit. Green halos around lenticels were the first GS symptoms visible at 47 days after full bloom (DAFB) (GHL stage). Green halos darkened with the onset of red coloring (stage GHGS). By 96 DAFB, GS symptoms began to turn brown and sink relative to the surrounding surface (BHL stage). The first macroscopically visible cracks appeared by 133 DAFB. Most GS (92.5%) were associated with lenticels. Frequency of GS decreased from the proximal stem end to the distal calyx end. The frequency of fruit with GHGS or BHL symptoms increased as fruit diameter increased (r^2^ = 0.36***, n = 66). Lenticels on bagged fruit did not develop GS. Thin sections through the skins within GHGS revealed coagulated, brownish cytoplasm and positive lignin staining. Epidermal cells were smaller, and cuticular ridges were thicker within GS symptoms than in peripheral asymptomatic peels. The frequency of cracked lenticels markedly increased from intact asymptomatic lenticels (OKL) to the final BHL stage (severe GS). The skin tension was highest for the GHGS and the BHL stages and lowest in bagged ‘WA 38’ (BL). Cuticle and wax mass per unit area were higher within a GS than outside of a GS area. Strain relaxation on wax extraction was largest for cuticles from GS. There was little relaxation on excision and isolation. Transpiration was significantly higher through skin segments with GHGS symptoms than those with OKL. In summary, skin characteristics within a GS on ‘WA 38’ resembled those of a severely strained fruit skin where the strain results from a localized restriction of surface growth. The skin characteristics of symptomatic apples must be considered a consequence rather than a cause of the GS disorder.

## Introduction

‘WA 38’ resulted from a cross of ‘Enterprise’ and ‘Honeycrisp’ and is marketed as Cosmic Crisp^®^ [1]. While ‘WA 38’ is known for its excellent consumption quality, an increasing number of reports on a novel “green spot" (GS) surface disorder have emerged in recent years [2–4]. This disorder is unknown in the parent cultivars, but other apple cultivars around the world show similar symptoms [5]. All have in common ‘Honeycrisp’ in their lineage [5]. The mechanistic basis of the GS disorder in ‘WA 38’ is not known, and effective countermeasures are lacking.

Green spot symptoms appear as green irregular patches, particularly on the proximal half of the fruit. Symptoms worsen, and patches become brown. In many cases, the fruit surface cracks macroscopically within a green spot. Often, lenticels are located in the center of the green spots. A wide range of cultural countermeasures were investigated, including training systems and pruning techniques, multiple spray applications of Ca salts, sprays of plant growth regulators, early overhead misting (Musacchi's unpublished data), netting, and phospholipid and polysaccharide-based coating [3], but none of them successfully suppressed GS. The only exception was preharvest bagging of fruit that consistently reduced or even prevented GS on ‘WA 38’ [3,4]. Unfortunately, the cost of labor for on-tree fruit-bagging is prohibitive and does not represent a feasible practice for the apple industry.

Macroscopic cracking of the fruit surface is associated with several surface disorders in apple. The formation of microscopic cracks precedes this macrocracking in the cuticle. When these microcracks traverse the cuticle, its barrier function is impaired, leading to physiological consequences [6]. During early fruit development, microcracking results in russeting, a disorder that compromises appearance [7,8]. Botanically, russeting is the formation of a periderm comprising a phelloderm, phellogen, and phellem [9]. The periderm is formed in the hypodermal cell layers [10,11]. The fractured cuticle is sloughed off. When the phellem reaches the surface, the suberin-encrusted cell walls of the phellem impart a brownish-dull appearance to the russeted surface. Late-developing microcracks in the cuticle result in skin spots, as the fruit peel has lost the ability to form a periderm [7,11–13]. Skin spots occur before or after harvest during storage. This skin spot disorder is caused by cell death underneath those microcracks that traverse the cuticle.

Whether microcracks also precede macrocracking of GS at or near lenticels in ‘WA 38’ is currently unknown. Such information, however, would help identify the etiology of GS formation in ‘WA 38’ and, ultimately, develop effective mitigation strategies.

The objectives of our study were to characterize green spot and establish the role of the cuticle in developing green spot on ‘WA 38’ fruit. In some experiments, the parental cultivars ‘Honeycrisp’ and ‘Enterprise’ served as comparisons. We used bagged ‘WA 38’ fruit as a positive control, as bagging consistently reduced or prevented symptom development [3].

## Materials and methods

### Plant material

Developing ‘WA 38,’ and its parents, ‘Enterprise’ (maternal contribution) and ‘Honeycrisp’ (paternal contribution) apples were sampled in 2024 from experimental orchards in the Washington State University Sunrise Research Orchard Station (SRO, Rock Island, WA, USA; 47°18'N, 120°07'W; elevation 267 m). ‘WA 38’ trees (planted in 2013) at SRO were grafted on G.41 rootstocks, while ‘Honeycrisp’ (planted in 2014) and ‘Enterprise’ (planted in 2008) trees were grafted on M9-T337.

At SRO, 608 ‘WA 38’ apples were bagged at 48 days after full bloom (DAFB) across 10 experimental trees (avg. 61 bags tree^-1^) grafted on G.41 and randomized across 5 rows. Each cluster was singularized by hand thinning to a single fruit per cluster before installing the bag. The same 2-layer apple bags were used as in our earlier study (Kobayashi Bag, Nagano, Japan) [3]. Another set of 10 ‘WA 38’/’G.41’ trees was also hand-thinned to single fruit and served as a comparison (659 total fruit, avg. 66 apples per tree^-1^). Bagging reduced or prevented green spot development in an earlier study and was therefore used as an additional control [3].

‘WA 38’ apples used for microscopy, assessing the depth of green spots, strain relaxation assays of cuticles, and quantifying the skin permeance to water vapor were sampled at maturity in 2023 at the Research Center, Laimburg, Province of Bozen, Italy (46°38'N, 11°29'W; elevation 220 m) and then shipped to the Leibniz University Hannover, Germany.

Tree fruit management was carried out in accordance with current regulations for integrated fruit production and standard practices in Washington State (USA) and Italy (Europe).

### Development of green spot – Macroscopy and microscopy

A total of 20 fruit of ‘WA 38’ were tagged in the orchard and photographed at regular time intervals beginning at 47 DAFB. Images of whole fruit and detailed images of lenticels were taken throughout the season (EOS Rebel T7 and EOS 70D DSLR, EF18–55 mm lens; Canon, Tokyo, Japan). Characteristic stages of development of lenticels with green spot were selected: intact lenticels (OKL), lenticels surrounded by a green halo (GHL), lenticels showing an early stage of green spot formation (GHGS) and lenticels that had green spots associated with necrosis as indicated by tissue browning (BHL). At selected time intervals (118 and 125 DAFB) a second set of apples were sampled from “control” and “bagged” ‘WA 38’/’G.41’ trees and transported to the laboratory for microscopic inspection. Lenticels from bagged fruit were included as an additional control (BL). The fruit were sampled into a cooler, brought to the laboratory within 2 h, rinsed using deionized water to remove adhering dust, and processed on the same day. The temperature of the cooler was 4°C. The data from the two sampling times were pooled. The minimum number of lenticels for each stage was 35. Fruit having lenticels representative of the BL, OKL, GHL, GHGS and BHL stages were transferred to the stage of a stereomicroscope (SMZ18 stereomicroscope; Nikon, Tokyo, Japan), viewed in incident white light and photographed (DS-Fi3 microscope camera; Nikon). The open pore area of the lenticel and the area of any microcracks traversing the lenticel were quantified by image analysis (NIS-Elements BR 5.30.03 software, Nikon; see S1 Fig for an example). Subsequently, the selected portion of the apple was incubated in a 0.1% (w/v) solution of the fluorescent tracer acridine orange containing the silicone surfactant Silwett^®^ L-77 (PhytoTechnology Laboratories^®^, Shawnee, KS, USA) at 0.05% for 10 min [14]. Earlier studies established that the silicone surfactant was needed to infiltrate lenticels and any cracks associated with lenticels. The fluorescent tracer acridine orange does not penetrate an intact cuticle, but in presence of a silicone surfactant, it penetrates all openings that bypass the cuticle. These include lenticels, microcracks, or stomata [15]. Following removal of the fruit from the dye solution, the surface was rinsed with deionized water for 30 sec and then blotted using soft tissue paper. An epidermal skin disc (ES, thickness 1–2 mm) with the lenticel of interest was excised using a biopsy punch (8 mm diameter; model 21909–146; Integra™ Miltex^®^, Avantor, Allentown, PA, USA) and a razor blade. ES disk comprised cuticle, epidermis, hypodermis, and adhering parenchyma. The ES were placed on moist filter paper in a petri dish, transferred to the microscope stage again (SMZ18 stereomicroscope, equipped with a P2-EFLC EGFP LP HC filter, emission wavelength 515–555 nm), and now viewed in incident fluorescent light. The fluorescing area surrounding an infiltrated lenticel was quantified using image analysis (NIS-Elements BR 5.30.03 software, Nikon). This area is a measure of the amount of tracer penetrated and hence, of the degree of opening of the lenticel [14].

### Lignin deposition

Cross sections from the skin of mature ‘WA 38’ were prepared by hand using a razor blade and investigated for lignin deposition. Briefly, lignin deposition in the cell wall was identified by incubating skin sections for 10 min in 2% (w/w) phloroglucinol prepared in 95% (v/v) aqueous ethanol. After adding a droplet of concentrated H_2_SO_4_, specimens were viewed in transmitted white light (BX-60; Olympus, Tokyo, Japan). A purple reddish color is indicative of lignin deposition in the cell wall when viewed through transmitted white light [13].

### Epidermal cell density, cell size, and thickness of anticlinal ridges

Scanning electron micrographs of the inner surface of cuticles isolated from healthy asymptomatic fruit, from symptomatic mature ‘WA 38’ within and outside of a green spot were prepared using a Quanta 200 scanning electron microscope (SEM) (FEI Europe Main Office, Eindhoven, The Netherlands). The cuticle isolation protocol is outlined below. Specimens were mounted on aluminum stubs using double-sided carbon tape and sputter-coated with gold. Calibrated images were prepared at 240× and an acceleration potential of 8 kV. The number of epidermal cells per unit fruit surface area and the width of anticlinal cuticular ridges between two adjacent epidermal cells were determined (Software Cell^P, Olympus Deutschland, Hamburg, Germany). The average size of epidermal cells was calculated by dividing the number of epidermal cells per image by the area of the image. Six replicates were used to estimate cell counts, and cell density ranged from 202 to 425 across the width of anticlinal cuticular ridges.

### Shape of green spots

At 118 and 125 DAFB, ‘WA 38’ fruit were collected at SRO and selected for GS in the GHGS and BHL category. Photographs were taken and the longitudinal length (parallel to the calyx stem cavity axis) and the latitudinal length (perpendicular to the calyx stem cavity axis) measured using image analysis (NIS-Elements software, Nikon). The ratio of longitudinal to latitudinal length was calculated. The total number of GS analyzed was 69.

### Depth of green spots

Mature ‘WA 38’ from the Research Centre Laimburg with green spots of the GHGS stage were transferred to a digital microscope (VHX-7000; Keyence, Osaka, Japan). A 3D-model of the cap of a sphere with a green spot in the center was selected to account for the curvature of the fruit surface. A surface scan was prepared at ×20–40. To quantify the depth of the green spot (GS) area, the curved 3D model of the fruit surface was flattened. Mean depths were then calculated for each of 40 transects—spaced 20 µm apart and with a minimum length of 1 mm—both within and outside the GS region. The mean depth of each green spot was calculated, with a healthy area outside the symptomatic region on the same fruit serving for comparison. Using this procedure, a total of 30 GS were investigated.

### Distribution of green spots on fruit surface, effect of fruit size

At 96 DAFB, the first BHL symptoms of GS in ‘WA 38’ developed at SRO. At this time, green spot frequency, most of which was associated with lenticels, was established on a total of 40 fruit. In addition, the distribution of GS in different regions of the fruit surface was quantified at 105 DAFB. The frequency of green spots in the distal calyx third, the equatorial third, and the proximal stem end third of ‘WA 38’ was established on a total of 37 fruit.

The effect of fruit size on the frequency of mature ‘WA 38’ apples with severe GS (stages GHGS and BHL) was established. Fruit from 8–10 trees were harvested at 159 DAFB, held in cold storage at 1°C for 35 days, then sorted into classes according to equatorial diameter. The total number of fruit and the number of fruit having severe green spots (GS classes GHGS and BHL) were quantified. Fruit exhibiting these symptoms would be excluded from marketing.

### Effect of green spots on skin tension as indexed by a gaping assay

The tension in the skin represents the driving force for the formation of cracks in the fruit skin [5,16,17]. Since green spots at the BHL stage are often associated with cracks [3], the skin tension was quantified in mature ‘WA 38’ at 133 DAFB using a gaping assay [16,17]. Ten apples were selected for the BL, OKL, GHL, GHGS, and BHL stages. Incisions were made on a total of 50 apples by cutting the skin in the stem end third with a razor blade to a depth of 5 mm and a cut length of 37 mm. Two cuts were made per fruit: a longitudinal cut parallel to the calyx-stem axis and a latitudinal cut perpendicular to the longitudinal cut. Subsequently, fruit were incubated in a closed plastic box above deionized water for approximately 22–24 hours. After that time, strain relaxation has long reached an equilibrium. Each box contained one apple for each stage of GS development. The water vapor saturated atmosphere inside the box prevented transpiration of the fruit, which could have resulted in artifacts. The apples were then placed on the stage of a stereomicroscope (SMZ18; Nikon), and the width of the gaps was quantified using image analysis (NIS-Elements BR 5.30.03 software; Nikon). The number of replicates was ten for each GS stage.

### Cuticle deposition

Cuticular membranes (CMs) were isolated enzymatically following the procedure by Orgell [18]. Epidermal skin discs were excised using a biopsy punch (8 mm diameter, 2 discs per fruit up to 75 DAFB; from 82 DAFB onwards 10 mm diameter, 3 discs per fruit) (Integra™ Miltex^®^, Avantor, Allentown, PA, USA). Unless otherwise specified, the ES were excised from the equatorial region of the apples. The discs were incubated in 50 mM citric acid buffer solution (pH 4.0) containing pectinase from *Rhizopus* spp. (40 g L^-1^; Millipore Sigma, Rockville, MD, USA) and cellulase from *Aspergillus niger* (4 g L^-1^; Millipore Sigma) and 30 mM sodium azide (NaN_3_). After shipping the sample to Hannover, Germany, the enzyme solution was replaced with 90 mL L-1 pectinase [Panzym Super E flüssig (Novozymes A/S, Krogshoejvej, Bagsvaerd, Denmark)] and 5 mL L^-1^ cellulase [(Cellubrix L.; Novozymes A/S)] in a buffered solution. Growth of microorganisms was suppressed by sodium azide at a final concentration of 30 mM. The enzyme solution was refreshed periodically. After the CMs were separated from the underlying tissue, the isolated CMs were carefully cleaned with a soft aquarelle brush, rinsed thoroughly with deionized water, dried individually on Teflon sheets, and then weighed on an analytical balance (CPA2P; Sartorius, Göttingen, Germany). Dewaxed CMs (DCMs) were prepared by incubating CM discs individually in 3 mL chloroform/methanol (1:1 v/v) overnight. After rinsing in chloroform/methanol, the DCMs that were obtained were dried and weighed individually. The amount of wax per unit surface area was calculated by subtracting the mass of DCM per unit surface area from that of the CM per unit surface area. All determinations were carried out with 20 replicates.

Using this procedure, the developmental time course of cuticle deposition was determined in ‘WA 38,’ ‘Honeycrisp,’ and ‘Enterprise.’

Cuticle deposition was compared between different regions of ‘WA 38’ apples. Fruit were sampled at 61, 89, and 159 DAFB. Due to the small size at 61 DAFB, ES were excised from calyx, equatorial and stem end region only. At 89 and 159 DAFB fruit ES were obtained from five regions, i.e., calyx, calyx-equatorial, equatorial, equatorial-stem, and stem end regions.

### Strain relaxation analysis

The releases of biaxial strain (ε all calculated in %) on excision of the ES (ε_exc_), isolation of the CM (ε_exc + iso_), and wax extraction (ε_extr_) were quantified as described previously [17]. Briefly, the ES were excised using a biopsy punch (10 mm diameter; Kai Europe, Solingen, Germany) and immediately submerged in silicone oil (AS 4; Wacker Chemie, Munich, Germany). After 24 h, a digital photograph was taken under a microscope (MZ10F; Leica Microsystem, Wetzlar, Germany; camera DP71, Olympus). The surface area of the ES (A_ES_) was quantified and calculated from the mean of two diameters of the ES measured perpendicular to each other (Software Cell^P, Olympus). The ε_exc + iso_ was determined on fully hydrated enzymatically isolated CM. The CM were spread on microscope slides. A calibrated digital image was taken (MZ10F; Leica Microsystem; camera DP71, Olympus). Two diameters of the CM disc were measured perpendicular to each other (Software Cell^P, Olympus). The area of the isolated and relaxed CM disc (A_CM_) was calculated. Subsequently, cuticular wax was extracted as described above. The DCM disc was hydrated, re-photographed and the area of the DCM disc quantified (A_DCM_). The A_Fruit_ refers to the area of the original disc before excision, i.e., the area of the biopsy punch used to excise the ES. The apparent releases of biaxial strain on excision of the ES ($\varepsilon_{exc}$; %), on excision and isolation of the CM ($\varepsilon_{exc+iso}$; %) and on wax extraction of the CM ($\varepsilon_{extr}$; %) were calculated as [19]:


$$\begin{array}{c} \varepsilon_{exc}=\frac{A_{Fruit}-A_{ES}}{A_{DCM}}\times \text{1}\text{0}\text{0} \end{array}$$ (1)

$$\begin{array}{c} \varepsilon_{exc+iso}=\frac{A_{Fruit}-A_{CM}}{A_{DCM}}\times \text{1}\text{0}\text{0} \end{array}$$ (2)

and


$$\begin{array}{c} \varepsilon_{extr}=\frac{A_{CM}-A_{DCM}}{A_{DCM}}\times \text{1}\text{0}\text{0} \end{array}$$ (3)

The total strain relaxation ($\varepsilon_{tot}$) was calculated as the sum of $\varepsilon_{exc+iso}$ and $\varepsilon_{extr}$.


$$\begin{array}{c} \varepsilon_{tot}=\varepsilon_{exc+iso}+\;\varepsilon_{extr} \end{array}$$ (4)

The ε_exc_, ε_exc + iso_, ε_extr_ and ε_tot_ were quantified in ‘WA 38,’ ‘Enterprise,’ and ‘Honeycrisp’ in the course of development, at maturity for asymptomatic and symptomatic ‘WA 38’ within and outside of a GS (stage GHGS) and for representative stages of GS development, i.e., the BL, OKL, GHL, GHGS, and BHL stages in ‘WA 38’ at 127 DAFB. Potential differences in strain relaxation on excision and isolation (ε_exc + iso_) along the calyx/stem axis were quantified in the calyx, the calyx equator, the equator, the equator stem end, and the stem end region of mature ‘WA 38.’ Because strain relaxation upon wax extraction was not quantified, the relaxation on excision and isolation was calculated as:


$$\begin{array}{c} \varepsilon_{exc+iso}=\frac{A_{Fruit}-A_{CM}}{A_{CM}}\times \text{1}\text{0}\text{0} \end{array}$$ (5)

### Effect of GS on water vapor permeance of fruit skin

The effect of GS on the water vapor permeance of the fruit skin of mature ‘WA 38’ fruit was studied. For this experiment, GS of the GHGS stage were selected because they had not yet been cracked. Epidermal skin segments were excised using a biopsy punch (10 mm diameter; Kai Europe, Solingen, Germany). The cut surface of the ES was blotted with tissue paper, and the ES was then mounted on stainless steel diffusion cells using a high-vacuum grease (Korasilon-Paste; Kurt Obermeier, Bad Berleburg, Germany; for a schematic drawing of the diffusion cell, see Knoche et al. [20]). A lid was placed on the diffusion cell so that the outer surface of the fruit skin was exposed in the orifice. The lid was sealed to the bottom using clear transparent tape (Tesa film; Beiersdorf, Norderstedt, Germany). Diffusion cells were filled with deionized water through a port in the bottom and the port was tape-sealed. The diffusion cells were held in a polyethylene (PE) box, upside down, above dry silica gel, with the skin surface facing the silica gel. The diffusion cells were equilibrated in this position overnight. The next day, diffusion cells were weighed, returned to the PE box, and reweighed at regular intervals. The flow rate of water across the fruit skin (*F*, kg s^-1^ per diffusion cell) was calculated as the slope of a linear regression line fitted through a plot of cumulative water loss per diffusion cell vs. time. The flow rates were then converted into flux densities (*J*, kg m^-2^ s^-1^) by dividing flow rates by the skin surface area exposed in the orifice of the diffusion cell (*A*_*orifice*_; m^2^).


$$\begin{array}{c} J=\frac{F}{A_{orifice}} \end{array}$$ (6)

The permeance of the fruit skin (*P*, m s^-1^) was then obtained by dividing the flux of water vapor across the fruit skin by the gradient in concentration of water vapor across the fruit skin (*ΔC*, kg m^-3^). This gradient in water vapor concentration represents the driving force for transpiration.


$$\begin{array}{c} P=\frac{J}{\Delta C} \end{array}$$ (7)

The gradient in concentration of water vapor equals the difference of water vapor concentration between the inside of the diffusion cell, i.e., the water vapor concentration at saturation (*c*_*i*_ = 19.44 g m^-3^ at 22 °C; [21]) and that outside of the diffusion cell above the dry silica gel (*c*_*o*_). Since the concentration of water vapor above dry silica gel is practically zero (*c*_*o*_ = 0 g m^-3^; [22]), the ΔC essentially equals *c*_*i*_.


$$\begin{array}{c} \Delta C=c_{i}-c_{o} \end{array}$$ (8)

Using this procedure, the skin permeance for water vapor of ES excised from symptomatic ‘WA 38’ (stage GHGS) and from asymptomatic ‘WA 38’ was calculated on an individual ES basis.

### Data analysis and statistics

The data in the figures and tables are presented as means ± standard errors of means. The only exception is Table 1, where medians are given. Where not shown in Figures, error bars are smaller than data symbols or data represent individual observations (i.e., Fig 4C; Fig 8, inset). Data were subjected to correlation analysis (Proc Corr), linear (Proc Reg) or non-linear regression analysis (Proc Nlin), analysis of variance (AOV; Proc GLM), or non-parametric tests (Proc Npar1way) using SAS (Version 9.1.4; SAS Institute, Cary, NC). Means were compared using Tukey's studentized range test at P = 0.05 or the Dunnett test at P = 0.05. Percentage data were arcsine transformed prior to AOV.

**Table 1:** Characteristics of development of lenticels on mature ‘WA 38’ as affected by green spots. The stages investigated were lenticels on bagged fruit (BL), or on non-bagged ‘WA 38’ normal lenticel (OKL), lenticel surrounded by green halo (GHL), lenticel with green halo and developing green spot (GHGS) and lenticel with fully developed green spot and associated cell death (BHL). The lenticels of bagged ‘WA 38’ were included because bagged fruit never developed green spots. The characteristics quantified were the frequency of lenticel with open pore, with crack, area of lenticel (pore plus crack) and necrotic area around lenticel. Fruit was incubated in a solution containing the fluorescent tracer acridine orange and a silicone surfactant and the infiltrated area around the lenticel area quantified by image analysis. For details, see text.

Stage	Frequency (%)		Area of lenticel pore plus crack	Necrotic area around lenticel	Area infiltrated by acridine orange
	Lenticel with open pore	Lenticel with crack	(µm^2^)	(mm^2^)	(mm^2^)
**BL**	34 b	9 b	0 ± 338 b	0 ± 0 b	0.47 ± 0.06 a
**OKL**	47 b	11 b	0 ± 164 b	0 ± 0 b	0.10 ± 0.03 b
**GHL**	86 a	39 b	335 ± 291 b	0 ± 0 b	0.14 ± 0.03 b
**GHGS**	95 a	35 b	565 ± 614 b	0.9 ± 0.2 b	0.09 ± 0.01 b
**BHL**	94 a	64 a	1290 ± 1136 a	7.8 ± 0.9 a	0.08 ± 0.02 b

Pooled data, n = 35–40

Non-parametric test by Proc Multtest and mean separation by contrast, P = 0.05

Data represent medians ± SE

## Results

### Development of GS and its distribution on the surface

**Fig 1:**
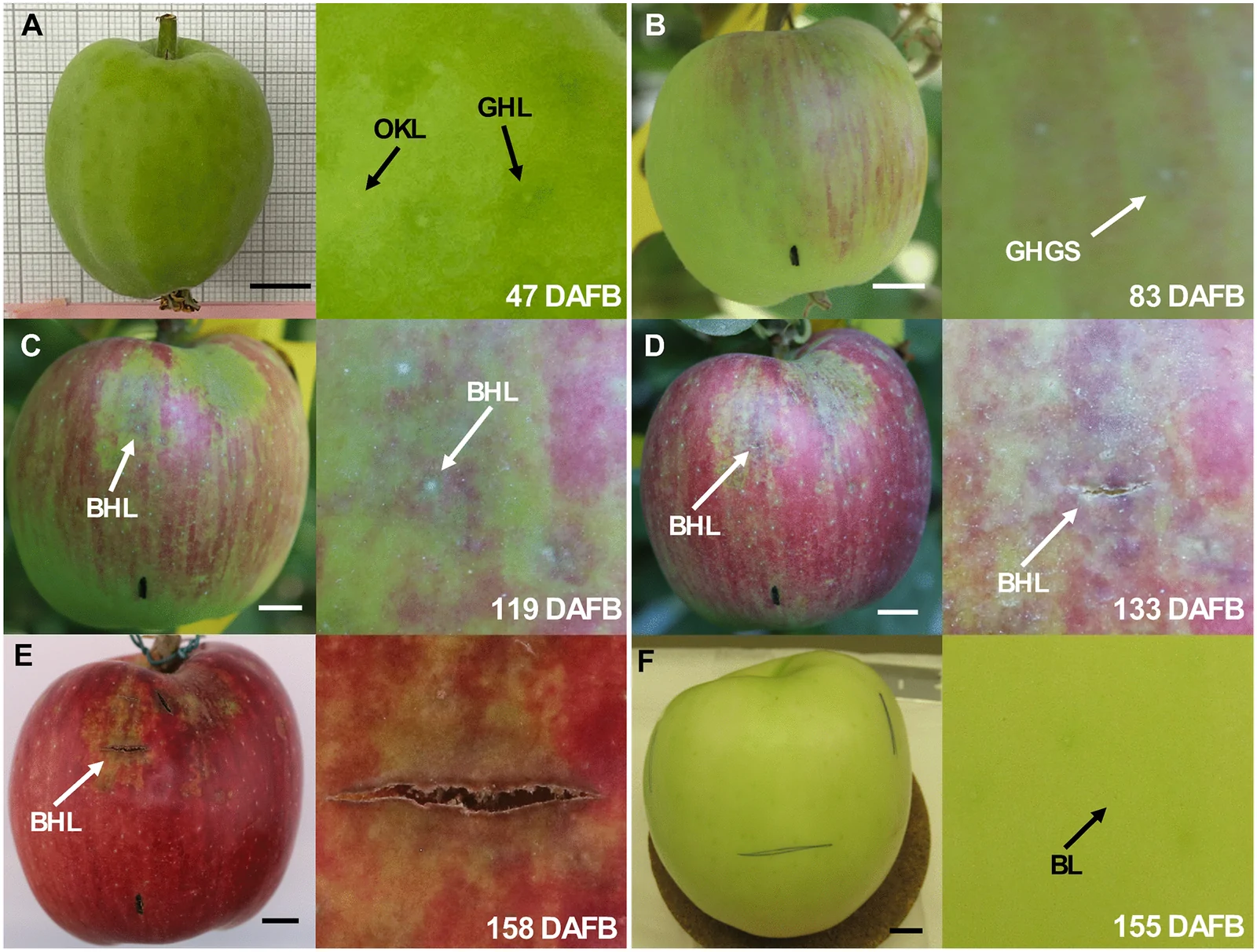
(A to E) Macroscopic and microscopic views of developing green spots of non-bagged ‘WA 38’ at different times. (F) Same as E, but picturing a bagged fruit. Time scale in days after full bloom (DAFB). Micrographs focus on lenticels that develop into green spots. Characteristic stages of green spot development from an intact lenticel (OKL) to a lenticel with green halo (GHL), a lenticel showing greening and initial green spot formation (GHGS) and a lenticel within a severe green spot that is associated with necrosis as indicated by skin browning (BHL). The first GHL occurred at about 47 DAFB, GHGS at 80 DAFB, and BHL at 96 DAFB. Arrows indicate typical stages of GS development. Images from B to E show the same fruit, with a lenticel developing from the GHL stage through GHGS to BHL. Scale bars in A to F are 10 mm.

The first signs of green spot development on ‘WA 38’ were observed at 47 DAFB (Fig 1A). Surrounding some lenticels, primarily in the stem end region of the fruit, green halos, somewhat darker than the surrounding surface (GHL stage), developed. Green halo areas increased as fruit continued to develop. Halo then turned darker green as the surrounding fruit surface began to color red (Fig 1B and 1C). This stage of GS development is referred to as the GHGS stage. At GHGS, the fruit surface was sunken relative to the surrounding surface. A representative example is shown in Fig 1B. By 109 DAFB, the first green spots had darkened and began to turn brown. This stage is referred to as the BHL stage. The extension of green spots was primarily longitudinal as indexed by a mean ratio of longitudinal length/latitudinal length of 1.15 ± 0.02 (mean for GHGS and BHL, n = 69, P = 0.001) (Fig 1C and 1D). By 133 DAFB, the first visible cracks appeared (Fig 1D and 1E). These macrocracks were oriented perpendicular to the calyx/stem end axis and usually traverse the lenticel in the center of the green spot. By 158 DAFB (harvest), a significant portion of symptomatic apples had developed severe cracks surrounded by necrotic tissue severe enough to downgrade or cull the apple (Fig 1E). Lenticels on bagged fruit did not develop green spots (Fig 1F). These are referred to as BL.

At 96 DAFB, about 92.5% of the green spots were associated with lenticels, and 7.5% were unrelated to lenticels. Green spots occurred most frequently in the proximal stem end third of the fruit (73.0%), less often in the central equatorial portion (27.0%) and rarely in the distal calyx part of the fruit.

The frequency of mature fruit that had severe GS symptoms (GHGS or BHL; ‘Freq’) increased linearly as fruit diameter increased (S2 Fig). The regression equation for this relationship was: Frequency (%) = 1.59 (±0.26) × diam (mm) – 86.57 (±19.94) (r^2^ = 0.36***, n = 66).

### Lenticel pore area and crack areas

**Fig 2:**
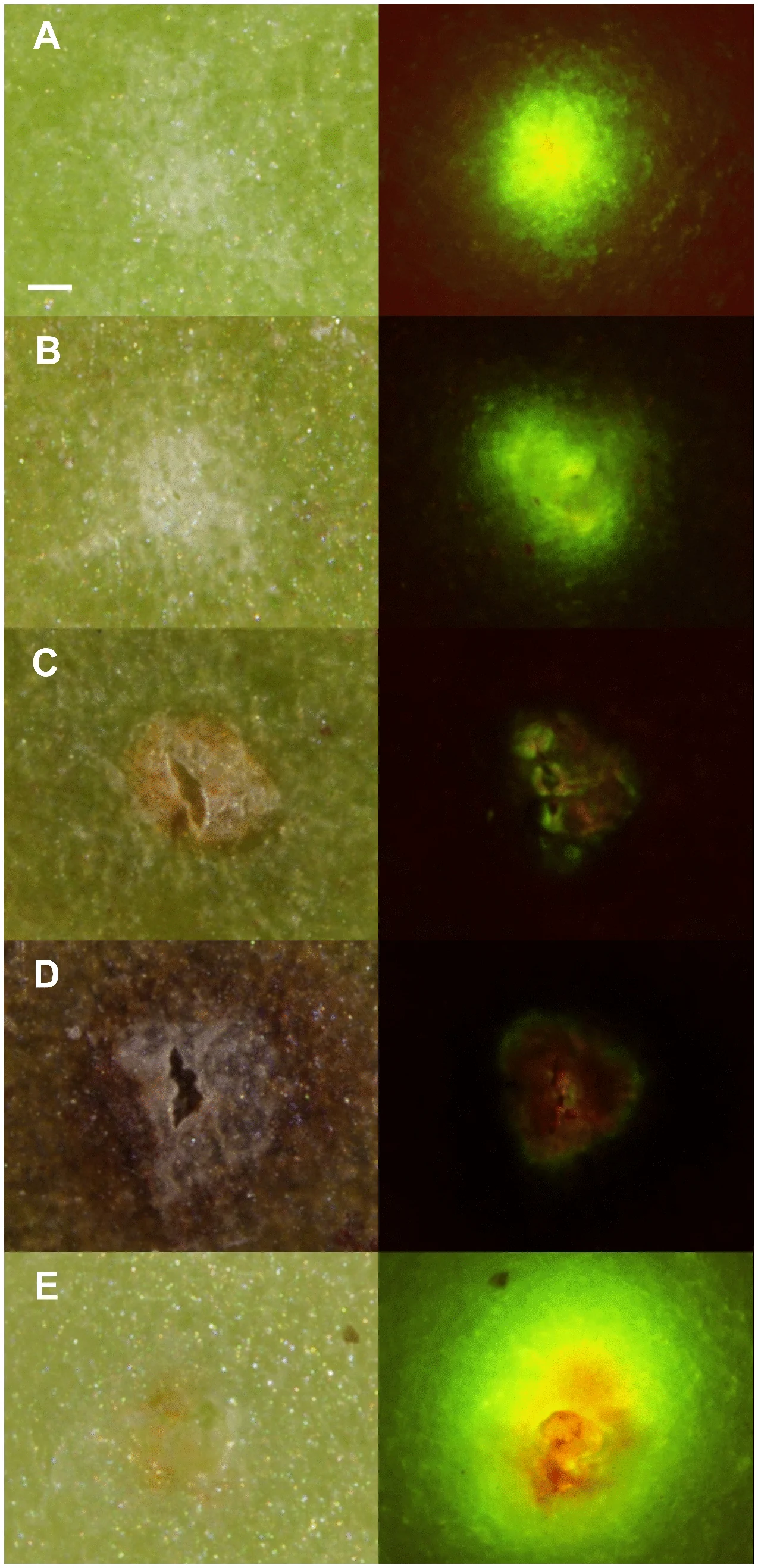
Micrographs of lenticels of ‘WA 38’ showing different stages of green spot development when viewed in incident white light (A-E) and fluorescent light following infiltration with acridine orange and a silicone surfactant. (A) lenticel without damage (‘OKL’), (B) lenticels with green halo (‘GHL’), (C) initial stage of green spot formation (‘GHGS’) and (D) green spot associated with necrosis as indicated by skin browning (‘BHL’). Lenticels from bagged ‘WA 38’ (‘BL’) in incident white light (E) and fluorescent light serve as a comparison. Bagged fruit never develops green spots. Scale bar is 1 mm.

The frequency of lenticels that had a microscopically visible open pore was lowest for the BL lenticels of the bagged fruit and increased from the OKL to the BHL stage for the non-bagged fruit (between 118 and 125 DAFB). There was no difference in open pore frequency between the OKL and the BL stages (Table 1; Fig 2). Also, the frequency of lenticels that were associated with microcracks markedly increased from the OKL to the GHL stage and to the final BHL stage. Again, there was no difference between OKL and BL stages. The sum of the open lenticel pore area and associated microcracks increased as GS developed (Table 1). Tissue browning, indicating necrosis, was limited to the BHL stage of GS development (Figs 1 and 2, Table 1). The infiltrated fluorescing area was largest in the BL lenticels of the bagged fruit. There was no significant difference in infiltrated area among OKL, GHL, GHGS and BHL stages (Table 1). The fruit surface between lenticels was smooth and without any microcracks in the cuticle (data not shown).

### Lignin deposition

**Fig 3:**
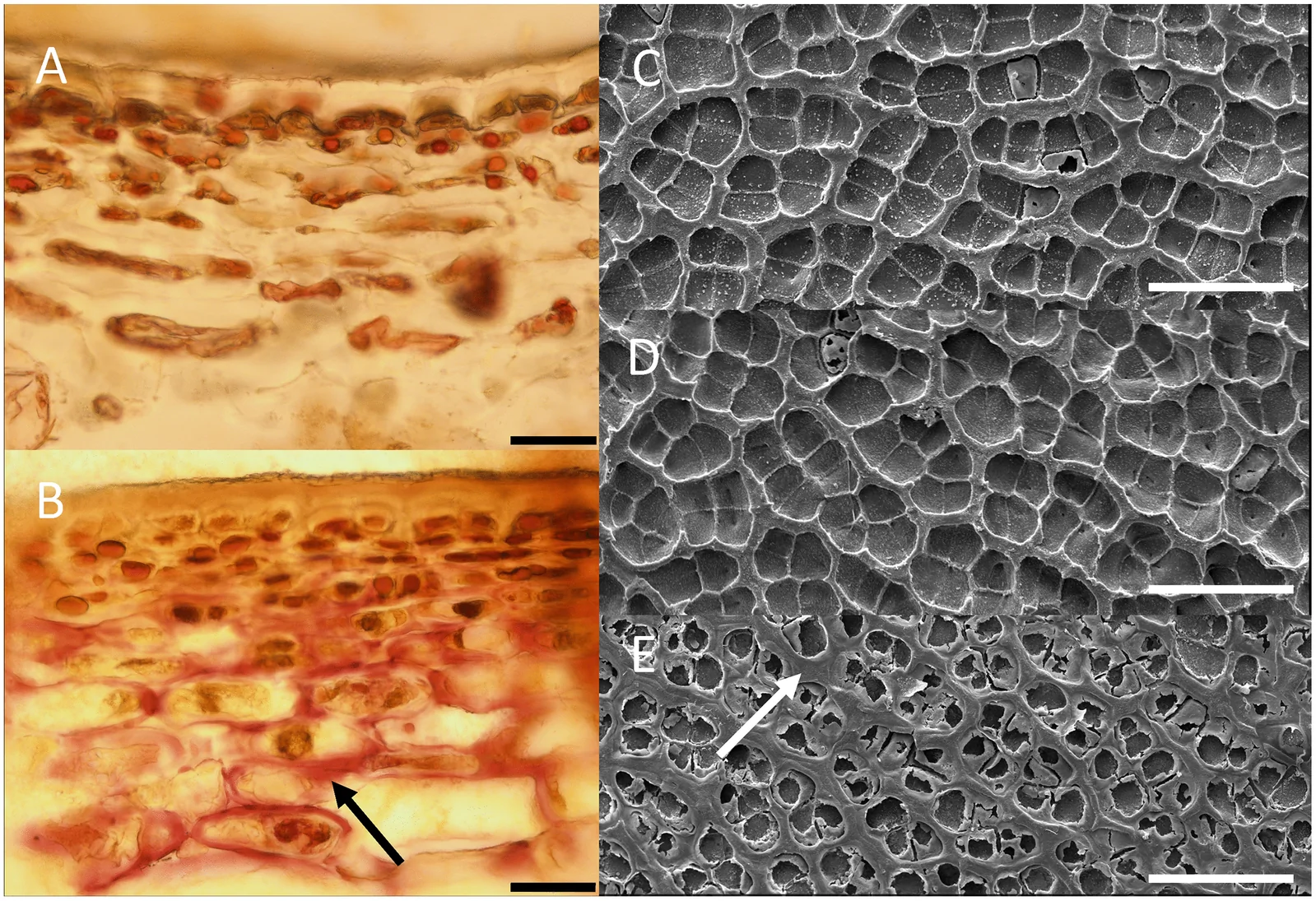
(A-B) Light micrographs of a cross section through a non-symptomatic healthy region (A) and through a green spot affected area (B) of a mature ‘WA 38’ fruit following lignin staining with phloroglucinol/H_2_SO_4_. Purple color in B (see arrow) indicates the presence of lignin at the boundary between a green spot and healthy tissue. Scale bar in A and B is 50 µm. (C to E) Scanning electron micrographs of the inner side of cuticles isolated from mature asymptomatic ‘WA 38’ (C) and symptomatic ‘WA 38’ outside of a green spot area (D) and within a green spot affected area (E). The network of anticlinal ridges of the cuticle between epidermal cells shows smaller cells and greater thickness of ridges in the green spot affected area as compared to the non-affected control. See arrow in E. Scale bar in C to E is 100 µm.

Thin sections through the fruit skin within green spots on mature ‘WA 38’ revealed cell collapse at the GHGS/BHL transition as indexed by a coagulated brownish cytoplasm and positive purple staining of cell walls with phloroglucinol/H_2_SO_4_ (Fig 3A and 3B). There was no such staining of lenticels at the OKL stage. Staining with phloroglucinol/ H_2_SO_4_ is indicative of lignin encrustation of cell walls. The lignin encrustation separated areas affected by green spot from non-affected areas of healthy asymptomatic tissue (Fig 3A and 3B).

### Epidermal cell size, density, and cuticular ridges

Epidermal cells within the GS were smaller and occurred at a higher number per unit area than those outside of the GS on the same fruit or those on a non-symptomatic fruit (Fig 3C-3E; Table 2). The cuticular ridges surrounding the imprints of epidermal cells were significantly thicker within the GS as compared to those of the asymptomatic control or outside of the GS on symptomatic fruit (Table 2, Fig 3C-3E).

**Table 2:** Number of epidermal cells per unit area, size of epidermal cells, and thickness of cuticular ridges between epidermal cells in asymptomatic ‘WA 38’ (’Asymptomatic fruit’), ‘WA 38’ with symptoms outside of the green spot (’Symptomatic fruit, outside of GS’) and within a GS (Symptomatic fruit, within GS). N = 6 for number of cells per unit area and for cell size; n = 220 to 415 for thickness of cuticular ridges. The GS selected here was at GHGS stage. For details see Materials and methods.

Category	Number of cells (No mm^-2^)	Cell size (µm^2^)	Thickness of cuticular ridges (µm)
**Asymptomatic fruit**	1197 ± 40 a	840 ± 29 a	6.5 ± 0.2 a
**Symptomatic fruit, outside of GS**	1343 ± 48 a	749 ± 26 a	7.0 ± 0.2 a
**Symptomatic fruit, within GS**	1598 ± 58 b	630 ± 24 b	9.0 ± 0.2 b

Data represent means ± SE. Mean separation by Tukey's studentized range test, P = 0.05.

### Depth of GS

**Fig 4:**
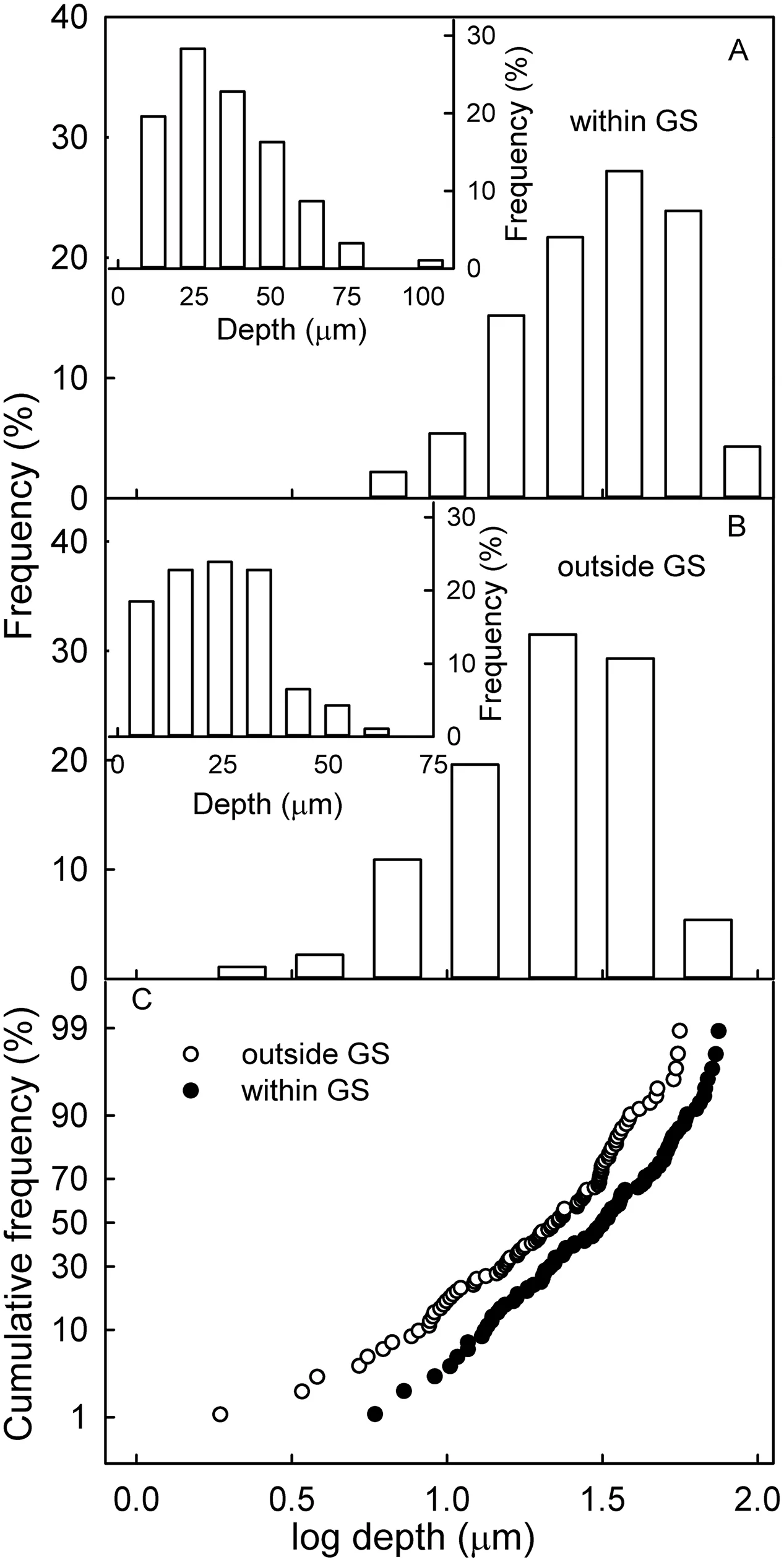
Frequency distribution of the depth of the rugosities in the surface of mature ‘WA 38’ fruit outside of a green spot (A) and within a green spot (B). (C) Log normal probability plot of cumulative frequencies of depth of rugosities in a healthy (‘outside GS’) and symptomatic area (‘within GS’).

The skin surface of ‘WA 38’ within and outside of green spot was not smooth, but rough on a microscopic scale. The distribution of the log depth of rugosities was normal both within and outside of a GS (Fig 4A and 4B). On average, the surface within a green spot was depressed by about 10 µm compared to that outside of a green spot (Fig 4C).

### Fruit growth, cuticle deposition, and strain relaxation within green spot

**Fig 5:**
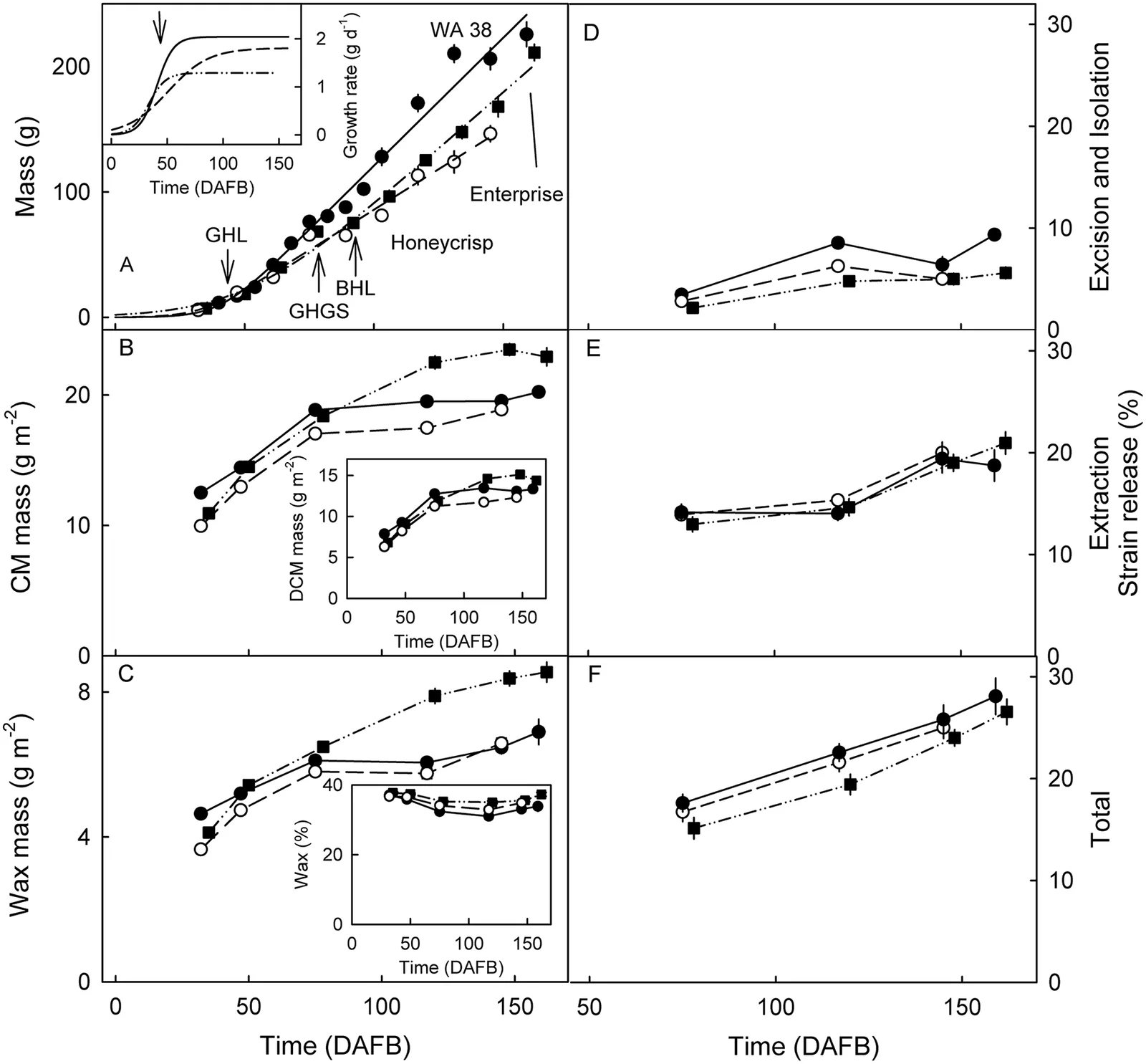
(A) Time course of fruit growth of ‘WA 38’ (black circles), ‘Honeycrisp’ (white circles), and ‘Enterprise’ (black squares). Insert: Fruit growth rate over time calculated as a first derivative of the expo linear regression model fitted through cumulative fruit mass vs. time. Arrows in A indicates time of first symptoms of lenticels with green halo (GHL) at 47 DAFB, of developing green spots (GHGL) at 80 DAFB and of a severe green spot that is associated with necrosis (BHL) at 96 DAFB in ‘WA 38.’ (B and C). Time course of the deposition of the cuticular membrane (CM) (B), the dewaxed CM (DCM) (B, insert) and epicuticular and cuticular wax (C). Insert in C: Change of wax percentage of the CM with time. X-axis scale in days after full bloom (DAFB). (D) Strain release upon excision and isolation of CMs (ε_exc + iso_), (E) after wax extraction (ε_extr_) and (F) the total strain release (ε_tot_) of CMs isolated from developing ‘WA 38,’ ‘Honeycrisp,’ and ‘Enterprise.’ The ε_tot_ was calculated as the sum of ε_exc + iso_ and ε_extr_. Data represent means ± SE.

Fruit mass of ‘WA 38’ increased after an initial lag phase nearly linearly with time (Fig 5A). The growth rate was highest in ‘WA 38,’ followed by ‘Enterprise’ and ‘Honeycrisp’ (Fig 5A, inset). The mass of the CM and the DCM per unit surface area increased up to 80 DAFB (Fig 5B, main graph and insert). It then remained fairly constant for ‘WA 38’ and ‘Honeycrisp,’ but continued to increase at a lower rate for ‘Enterprise.’ The mass of wax per unit area followed the same pattern in all three cultivars (Fig 5C), the percentage of wax remained constant. Statistical analyses revealed that CM, DCM, and wax mass were highest in ‘Enterprise’ followed by ‘WA 38’ and ‘Honeycrisp.’ Strain release upon excision and isolation of the CM, upon wax extraction and total strain release increased in the course of development (Fig 5D-5F). Strain release on excision and isolation was larger in ‘WA 38’ and ‘Honeycrisp’ than in ‘Enterprise’ (Fig 5D). There was no difference in strain release after wax extraction or in total strain release (Fig 5E-5F).

The masses of CM, DCM, and wax did not differ significantly between regions along the calyx stem end axis, whereas the strain release on excision and isolation was highest in the equatorial region and decreased towards calyx and stem cavity (S1 Table). Similar to the previous experiment (Fig 5D), strain relaxation on excision and isolation was highest in ‘WA 38,’ followed by ‘Honeycrisp’ and by ‘Enterprise’ (S1 Table).

**Fig 6:**
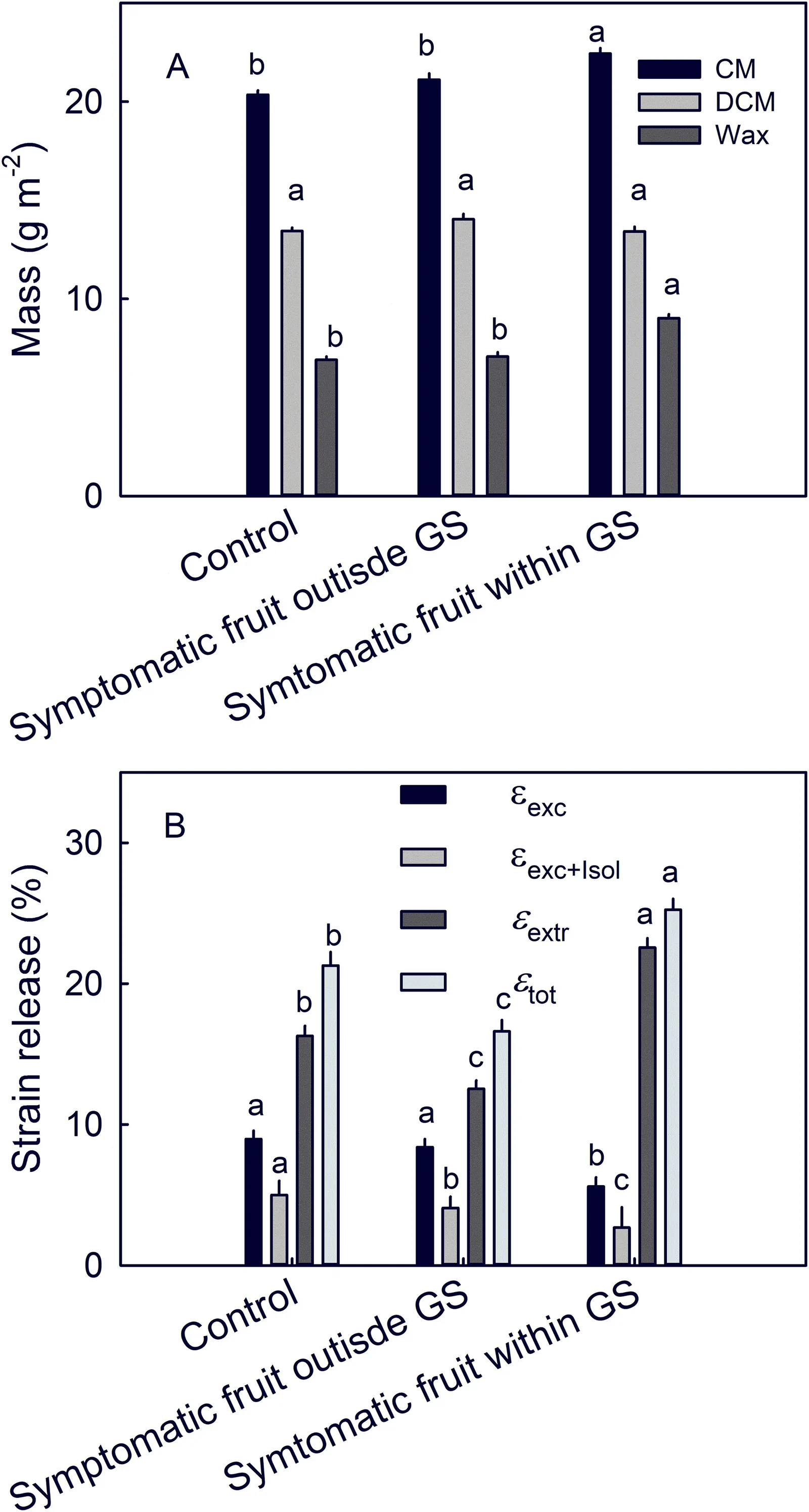
(A) Mass of the cuticular membrane (CM), the dewaxed CM (DCM), and the wax of asymptomatic and symptomatic mature ‘WA 38’ fruit outside and within a green spot. (B). Strain release of a skin disc following excision (ε_exc_), of the cuticle following excision and isolation (ε_exc+iso_), and following wax extraction (ε_extr_**).** For details, see the Materials and methods section.

Cuticle mass per unit area was higher within a GS as compared to non-affected areas on the same fruit or to asymptomatic ‘WA 38’ (Fig 6A). This was primarily due to increased wax deposition, while cutin deposition was unaffected. Strain relaxation on excision of the epidermal disc (ε_exc_), and on excision and isolation of the cuticle (ε_iso_) was less for discs within a GS than for those outside of a GS on a symptomatic fruit or from asymptomatic fruit (’control’) (Fig 6B). However, relaxation of the cuticle occurring upon wax extraction (ε_extr_) and total strain relaxation (ε_tot_) was greatest for cuticles isolated from GS (Fig 6B).

**Fig 7:**
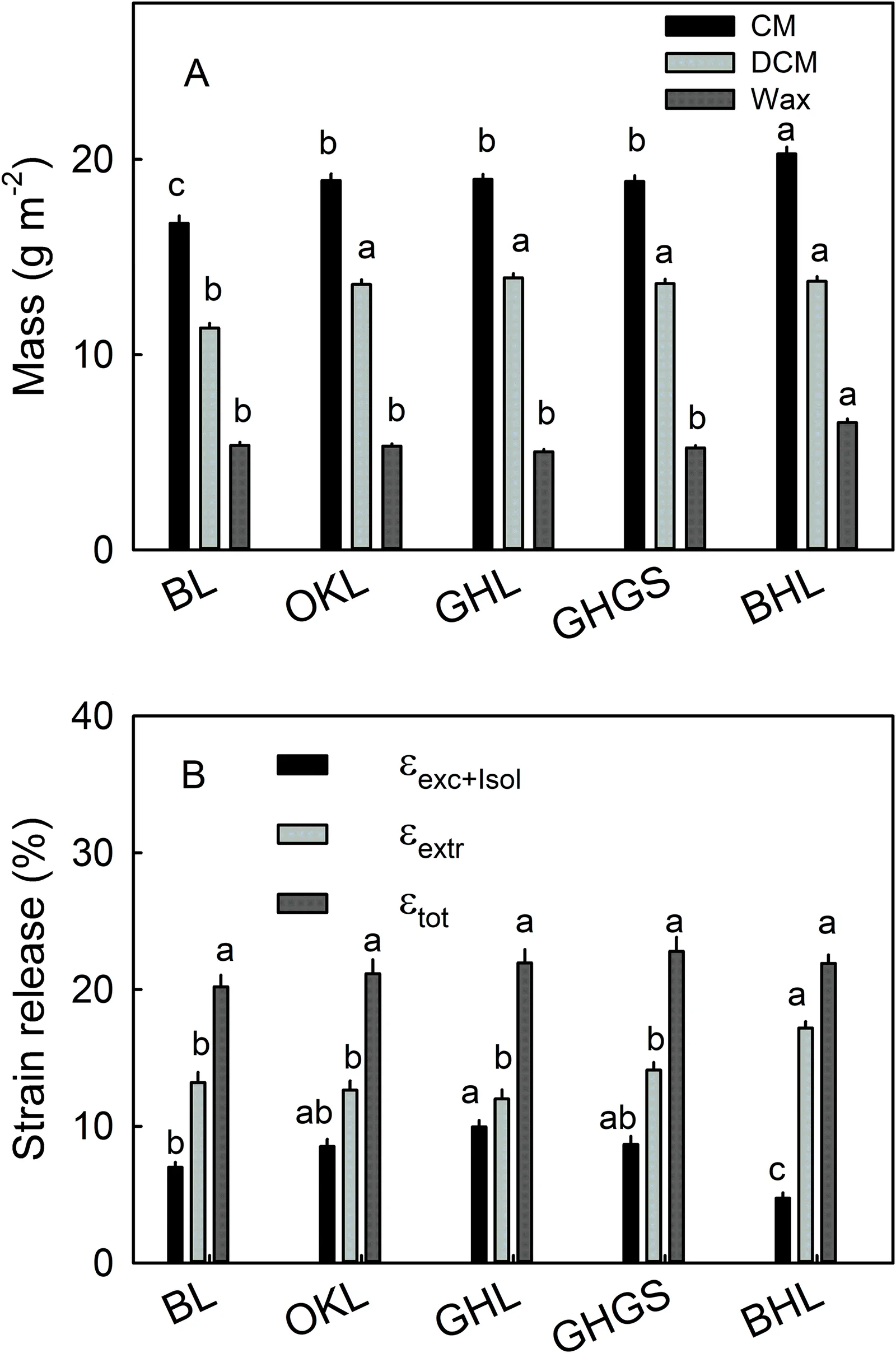
(A) Mass of the cuticular membrane (CM), the dewaxed CM (DCM) and wax and (B) strain release of cuticles excised from bagged fruit (BL), lenticels without damage (OKL), lenticels with green halo (GHL), initial stage of green spot (GS) formation (GHGS), and green spot associated with necrosis as indicated by skin browning (BHL). All samples were taken from the stem cavity region 127 days after full bloom, when all stages of GS development were present. Strain release was quantified after excision and isolation of the CM (ε_exc + iso_) and after wax extraction (ε_extr_). Total strain release (ε_tot_) was calculated as the sum of ε_exc + iso_ and ε_extr_.

Fruit that were bagged had lower CM and DCM mass per unit area than non-bagged fruit. There was no difference in CM mass, DCM mass, or wax mass between OKL, the GHL and the GHGS stage of GS development, but GS of the BHL stage had the highest CM and wax mass per unit area (Fig 7A).

Strain release on excision and isolation, and that on wax extraction, were significantly higher for GS of the BHL stage than for the OKL, GHL, and GHGS stages (Fig 7B).

### Gaping assay

As indicated by gaping upon incision, skin tension increased as GS development progressed (Table 3). The highest tension was associated with the GHGS and the BHL stages. Interestingly, the lowest skin tension was always associated with bagged ‘WA 38’ (BL) as compared to non-bagged fruit (Table 3). The tension in the latitudinal direction, i.e., perpendicular to the calyx/stem axis, was always larger than in the longitudinal direction, i.e., parallel to the calyx/stem axis (Table 3).

**Table 3:** Tissue tension as affected by the stage of green spot (GS) development on ‘WA 38.’ The tissue tension was quantified as the width of a gape formed upon incisions of the fruit skin of mature ‘WA 38’ with a razor blade. Stages of GS development (Fig 1) were normal lenticels on bagged ‘WA 38’ (BL), on non-bagged ‘WA 38’ normal lenticel (OKL), lenticel surrounded by green halo (GHL), lenticel with green halo and developing green spot (GHGS), and lenticel with fully developed green spot and associated cell death (BHL). The BL were included as controls because ‘WA 38’ bagged early in its development and did not develop green spots.

Stage	Gape width (mm)		
	Perpendicular to stem/calyx axis	Parallel to stem/calyx axis	Mean
**BL**	0.21 ± 0.03	0.17 ± 0.02	0.19 ± 0.02 c
**OKL**	0.59 ± 0.04	0.53 ± 0.03	0.56 ± 0.03 b
**GHL**	0.64 ± 0.04	0.55 ± 0.03	0.59 ± 0.03 b
**GHGS**	0.83 ± 0.04	0.72 ± 0.04	0.78 ± 0.03 a
**BHL**	0.79 ± 0.04	0.76 ± 0.05	0.78 ± 0.03 a
**Mean**	0.61 ± 0.04 a	0.55 ± 0.03 b	

Data represent means ± SE. Two factorial analysis of variance revealed significant main effects for stage and gape orientation, but no interaction. Mean separation within main effects by Tukey's Studentized range test, P = 0.05.

### Transpiration assay

**Fig 8:**
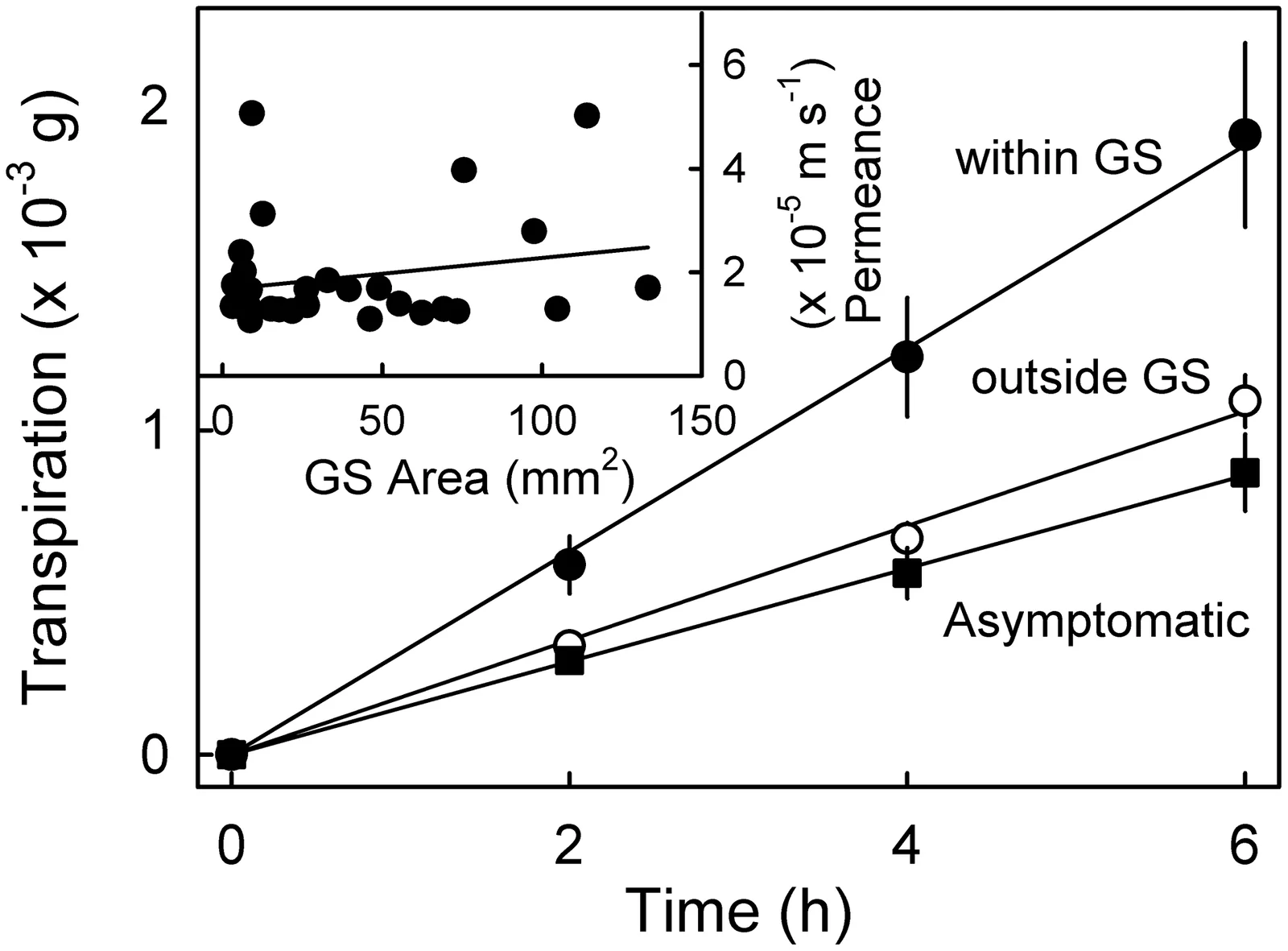
Time course of transpiration through epidermal skin segments (ES) excised from mature asymptomatic (asymptomatic) and symptomatic ‘WA 38’ fruit within (within GS) and outside of a green spot (GS; outside GS) affected area. Insert: Relationship between the area of the GS and the water vapor permeance of the ES.

Water vapor loss through skins of mature ‘WA 38’ increased linearly with time, indicating that flow rates were constant (Fig 8). Flow rates and skin permeances for water vapor was significantly higher for ES with green spots (stage GHGS), as compared to ES from area without GS in symptomatic fruit or those from asymptomatic control fruit. The water vapor permeance (P) of the skin was independent of the size of the GS (P (x 10^−5^ m s^-1^) = 2.002 (± 0.340) + 0.007 (0.006) × area (mm^2^), r^2^ = 0.04, n = 30; Fig 8, inset).

## Discussion

### Symptoms of green spot in ‘WA 38’ are similar to those of bitter pit in ‘Honeycrisp’

The symptoms of GS in ‘WA 38’ were similar to those of bitter pit (BP) in ‘Honeycrisp.’ In the early stages, both GS and BP exhibit water-soaked dermal tissue [23–25]. This water soaking resulted in the ‘green halo’ classification of lenticels (GHL stage) in our study. Water soaking is caused by flooding of the gas-filled intercellular cell wall space. Flooding occurs when membrane integrity is impaired, for example, due to localized Ca deficiency. Ca occurs in various pools within the plant. The pool of apoplastic free Ca is important for binding to membranes and for maintaining membrane integrity [23,26]. Accordingly, Ca deficiency causes membrane leakage [23,24].

The distribution of GS and BP along the stem/calyx axis slightly differed between GS and BP. Whereas the frequency of GS decreased from the proximal stem end to the distal calyx end of the fruit [3], the frequency of BP increased in the same direction [27]. All GS and most BP occur close to or at the surface [2,3,27]. The tissue browning and depressions of the fruit skin caused by BP were similar to those associated with GS.

Most GS symptoms were associated with lenticels, but no such findings were reported for BP. The association between GS and lenticels is not entirely clear. Lenticels represent sites of preferential transpiration [28]. It may be speculated that Ca is preferentially translocated to lenticels due to their high permeance to water vapor compared to that of the surrounding cuticle [28]. When the xylem ruptures due to fruit growth, the lenticels become uncoupled from the transpiration stream and from Ca transport in the xylem. This could lead to symptoms of deficiency, such as membrane leakage and water-soaking. Imura et al. [29] and Matsumoto et al. [30] reported that the xylem even connects to lenticels in ‘Kurenainoyume’ apples. However, no such connections were observed in ‘WA 38.’

Symptoms of GS in ‘WA 38’ occur exclusively preharvest [2,3], whereas those of BP in ‘Honeycrisp’ may develop preharvest (far after the inception of green spot) and postharvest [31]. The increase in BP incidence during postharvest period has been attributed to an internal redistribution of Ca from the cortex to the pith [32].

The above comparison shows that the symptoms of GS ‘WA 38’ and BP ‘Honeycrisp’ are broadly similar.

### Skin characteristics are typical of strained fruit skin

The changes in skin characteristics during GS formation are typical of a response of the fruit skin to tangential strain. The tangential strain results from locally impaired growth in surface area within GS symptoms while the fruit continues to grow, with the remaining asymptomatic surface later expanding. Impaired growth can be inferred from the smaller epidermal cells, increased cell density within the epidermis, and depression of the surface within symptomatic skin compared with skin outside of a GS. This makes GS symptoms a rigid island surrounded by an extensible sea. The continued increase in the surface area of the growing fruit subjects this skin to considerable tangential growth stress and strain. The stress focuses on the GS. Supporting evidence for this hypothesis comes from the following observations.

First, skin tension increased as GS development proceeded. Gaping occurs when the elastic portion of the skin strain relaxes [16,17]. The gape formed perpendicular to the direction of the strain. The gape was always larger perpendicular to the stem/calyx axis than parallel to it, indicating that growth of ‘WA 38’ caused higher growth stress and strain in the longitudinal than in the latitudinal transverse direction [16]. This is consistent with the growth pattern of ‘WA 38’ [33].

Second, anticlinal cuticular ridges of epidermal cells were thicker within a GS as compared to an adjacent area outside of the GS. Thickening of cuticular ridges is also a typical response to excessive tangential stress and strain of the skin [34]. During expansion, the thickening occurs when adjacent epidermal cells unbound along their anticlinal cell walls. Part of the anticlinal cell wall reorients and becomes a periclinal cell wall. The resulting void between the cells is filled with newly deposited cutin that forms the anticlinal ridges [34]. Unbounding of adjacent epidermal cells is consistent with the large tangential strain observed in the gaping assay and the smaller epidermal cells within the GS symptoms.

Third, the cuticle within a GS was thicker as indexed by a higher mass per unit area. The increase in thickness was due to an increased amount of wax. This finding also indicates high tangential growth stress and strain. Wax ‘fixes’ the strain within the expanding cutin polymer [35]. As a consequence, strain relaxation of an ES on excision or on excision and isolation of the cuticle was only small within symptomatic skin. However, when wax was extracted, the cuticle isolated from symptomatic skin relaxed much more than that of asymptomatic skin [35]. These data are also consistent with an increase in lipid compounds, such as triterpenes, showing elevated concentrations in the peel and cortex of GS-affected areas, and fatty acids within a GS-affected cortex (not in the skin) underlying the sunken GS area, as recently reported in a metabolomic study by Sheick et al. [4]. Marked tangential strain within a GS is also consistent with the large open pore area and the frequency of cracked lenticels in developing GS (Table 1).

Fourth, we observed lignin deposition in the cell wall at the boundary between a GS and the surrounding healthy tissue. Lignin deposition on the cell wall is a typical reaction that occurs between collapsing and healthy tissue, in an attempt by the healthy tissue to restore the impaired barrier properties of the collapsing skin [6]. In apple, periderm formation, an alternative mode of restoring an impaired barrier function, is limited to early fruit development and the ability to form a periderm is lost in the course of development and – most likely – at the stage the skin cracks in GS formation [7,36]. Thus, ‘WA 38’ cannot seal off the wound by formation of a periderm but must respond by depositing lignin in the cell wall [6]. Lignin deposition is also common to other skin spot disorders of apple fruit [12,13].

Fifth, lenticel characteristics such as the frequency of open pores, the frequency of cracks associated with lenticels, and the area of the opening of the lenticel (pore plus crack) all increased as GS development progressed. Preferential cracking at lenticels and stomata has been reported earlier in other fruit crop species [14,37,38]. This phenomenon is caused by differences in elasticity between lenticels and the surrounding surface. As argued above, lenticels cause stress concentration and rupture.

Sixth, formation of a depression of the GS and the browning of the tissue around the lenticel are indicative of cell collapse and necrosis. Subsequently, water is lost by lenticel (and cuticular) transpiration. This interpretation is consistent with the increased water-vapor permeance of lenticels reported in this and earlier studies [28]. Those later stages of GS formation, i.e., GHGS and BHL, are not surrounded by fluorescing tissue in our acridine orange assay, must not be interpreted as a lack of infiltration due to plugging with wax. The more likely explanation is the lack of dye uptake into epidermal and hypodermal cells due to cell death. Mass flow through the open pore and the large cracks at the lenticel will certainly occur in the presence of the silicone surfactant [15]. The fluorescent tracer acridine orange does not fluoresce unless taken up by living cells. This explains the lack of fluorescence of GS at the GHGS and BHL stage.

These arguments demonstrate that the changes in skin characteristics of developing GS of ‘WA 38’ are consistent with a typical response to excessive tangential strain of the fruit skin resulting from impaired expansion of epidermal and possibly hypodermal cells surrounding a lenticel on rapidly growing fruit.

## Conclusion

Our data indicate that the symptoms of GS in ‘WA 38’ are similar to those of BP in ‘Honeycrisp.’ The similarity of symptoms and the close genetic relationship between the two cultivars suggest a common or similar mechanistic basis. In ‘Honeycrisp,’ the high susceptibility to BP has been linked to the rapid rate of fruit growth, which causes imbalances in Ca and hormone supply [39]. Whether this also applies to GS in ‘WA 38’ remains to be seen. Changes in the skin properties identified in the present study resemble a typical response of a fruit surface to high tangential strain. This strain, in turn, results from impaired surface expansion in the GS-affected area, possibly due to local Ca deficiency, on a rapidly growing fruit. Therefore, changes in skin properties are the consequence, but not the cause, of GS in ‘WA 38.’

## Supporting information

S1 Fig Representative micrographs of lenticels on ‘WA 38’ fruit. (A) Lenticel with open pore area (yellow trace). (B) Lenticel with a microcrack (red trace) that traverses the open pore area (yellow trace). For details see materials and methods.
(TIF)

S2 Fig Relationship between the frequency of mature ‘WA 38’ fruit with severe green spot (GS) symptoms (GHGS and BHL stages) and fruit diameter in the equatorial plane. Fruit were harvested 159 days after full bloom. For the description of stages of GS development see text. The regression equation was: Frequency (%) = 1.59 (±0.26) × diameter (mm) – 86.57 (±19.94) (r^2^ = 0.36***, n = 66). Frequency (%) = 1.59 (±0.26) × diameter (mm) – 86.57 (±19.94) (r^2^ = 0.36***, n = 66).
(TIF)

S1 Table Mass of the cuticular membrane (CM) and strain release on excision and isolation of the CM in mature ‘WA 38,’ ‘Honeycrisp,’ and ‘Enterprise’. (DOCX)

S1 Data This is the excel file containing all data used to generate tables and figures, including the figures of the supplemental information. (XLSX)

## References

[1] Evans KM, Barritt BH, Konishi BS, Brutcher LJ, Ross CF. ‘WA 38’ apple. horts. 2012;47(8):1177–9. doi: 10.21273/hortsci.47.8.1177

[2] Sallato B, Whiting MD, Munguia J. Rootstock and nutrient imbalance leads to ‘“Green Spot”’ Development in ‘WA 38’ apples. Horts. 2021;56(12):1542–8. doi: 10.21273/hortsci16213-21

[3] Sheick R, Serra S, Rudell D, Musacchi S. Investigations of multiple approaches to reduce green spot incidence in ‘WA 38’ apple. Agronomy. 2022;12(11):2822. doi: 10.3390/agronomy12112822

[4] Sheick R, Serra S, Musacchi S, Rudell D. Metabolic fingerprint of “WA 38” green spot symptoms reveals increased production of epicuticular metabolites by parenchyma. Sci Hortic. 2023;321:112257. doi: 10.1016/j.scienta.2023.112257

[5] Al Shoffe Y, Robinson T, Fazio G, Follett E, Clark M, Luby J, et al. “Honeycrisp”: the challenge of the apple crisp revolution. F. 2025;5(1):0–0. doi: 10.48130/frures-0025-0030

[6] Knoche M, Lang A. Ongoing growth challenges fruit skin integrity. Crit Rev Plant Sciences. 2017;36(3):190–215. doi: 10.1080/07352689.2017.1369333

[7] Winkler A, Grimm E, Knoche M, Lindstaedt J, Köpcke D. Late-season surface water induces skin spot in Apple. horts. 2014;49(10):1324–7. doi: 10.21273/hortsci.49.10.1324

[8] Chen Y-H, Straube J, Khanal BP, Knoche M, Debener T. Russeting in apple is initiated after exposure to moisture ends-i. histological evidence. Plants (Basel). 2020;9(10):1293. doi: 10.3390/plants9101293 33008020 PMC7650782

[9] Evert RF. Esau’s plant anatomy, meristems, cells, and tissues of the plant body: their structure, function, and development. 3rd ed. New Jersey: John Wiley & Sons; 2006. 10.1002/0470047380

[10] Meyer A. A study of the skin structure of “Golden Delicious” apples. J Amer Soc Hortic Sci. 1944;45:105–10.

[11] Simons RK, Chu MC. Periderm morphology of mature “Golden Delicious” apple with special reference to russeting. Sci Hortic. 1978;8(4):333–40. doi: 10.1016/0304-4238(78)90055-9

[12] Khanal BP, Knoche M. Skin spots on cripps pink and elstar apples are identical. J Appl Bot Food Quality. 2024;97:127–32. doi: 10.5073/JABFQ.2024.097.015

[13] Grimm E, Khanal BP, Winkler A, Knoche M, Köpcke D. Structural and physiological changes associated with the skin spot disorder in apple. Postharvest Biol Tech. 2012;64(1):111–8. doi: 10.1016/j.postharvbio.2011.10.004

[14] Peschel S, Knoche M. Characterization of microcracks in the cuticle of developing sweet cherry fruit. J Amer Soc Hort Sci. 2005;130(4):487–95. doi: 10.21273/JASHS.130.4.487

[15] KNOCHE M. Organosilicone surfactant performance in agricultural spray application: a review. Weed Research. 1994;34(3):221–39. doi: 10.1111/j.1365-3180.1994.tb01990.x

[16] Skene DS. Growth stresses during fruit development in Cox’s Orange Pippin apples. J Hortic Sci. 1980;55(1):27–32. doi: 10.1080/00221589.1980.11514897

[17] Grimm E, Peschel S, Becker T, Knoche M. Stress and strain in the sweet cherry skin. J Am Soc Hortic Sci. 2012;137(6):383–90. doi: 10.21273/JASHS.137.6.383

[18] Orgell WH. The isolation of plant cuticle with pectic enzymes. Plant Physiol. 1955;30(1):78–80. doi: 10.1104/pp.30.1.78 16654733 PMC540601

[19] Lai X, Khanal BP, Knoche M. Mismatch between cuticle deposition and area expansion in fruit skins allows potentially catastrophic buildup of elastic strain. Planta. 2016;244(5):1145–56. doi: 10.1007/s00425-016-2572-9 27469168

[20] Knoche M, Peschel S, Hinz M, Bukovac MJ. Studies on water transport through the sweet cherry fruit surface: characterizing conductance of the cuticular membrane using pericarp segments. Planta. 2000;212(1):127–35. doi: 10.1007/s004250000404 11219577

[21] Nobel PS. Physicochemical and environmental plant physiology. San Diego, CA, USA: Academic Press; 1999.

[22] Geyer U, Schönherr J. In vitro test for effects of surfactants and formulations on permeability of plant cuticles. ACS symposium series. American Chemical Society; 1988. p. 22–33. 10.1021/bk-1988-0371.ch003

[23] de Freitas ST, Amarante CVT do, Labavitch JM, Mitcham EJ. Cellular approach to understand bitter pit development in apple fruit. Postharvest Biol Tech. 2010;57(1):6–13. doi: 10.1016/j.postharvbio.2010.02.006

[24] de Freitas ST, Jiang C-Z, Mitcham EJ. Mechanisms involved in calcium deficiency development in tomato fruit in response to gibberellins. J Plant Growth Regul. 2011;31(2):221–34. doi: 10.1007/s00344-011-9233-9

[25] Fuller MM. Cell ultrastructure in apple fruits in relation to calcium concentration and fruit quality. Acta Hortic. 1980;(92):51–6. doi: 10.17660/actahortic.1980.92.6

[26] de Freitas ST, Mitcham EJ. Factors involved in fruit calcium deficiency disorders. horticultural reviews. Wiley; 2012. 107–46. 10.1002/9781118351871.ch3

[27] Ferguson IB, Watkins CB. Bitter pit in apple fruit. Horticultural reviews. Wiley; 1989. 289–355. 10.1002/9781118060841.ch8

[28] Khanal BP, Si Y, Knoche M. Lenticels and apple fruit transpiration. Postharvest Biol Technol. 2020;167:111221. doi: 10.1016/j.postharvbio.2020.111221

[29] Imura E, Nakagomi M, Hayashida T, Fujita T, Sato S, Matsumoto K. Unraveling the mechanism of cork spot-like physiological disorders in “kurenainoyume” apples based on occurrence location. Plants (Basel). 2024;13(3):381. doi: 10.3390/plants13030381 38337914 PMC10857259

[30] Matsumoto K, Kobayashi T, Kougo T, Fujita T, Sato S, Moriguchi T. Prevention of new cork spot-like physiological disorder in “Kurenainoyume” apples by pre-harvest fruit bagging. Hort J. 2018;87(2):174–83. doi: 10.2503/hortj.okd-117

[31] Rosenberger DA, Schupp JR, Hoying SA, Cheng L, Watkins CB. Controlling bitter pit in `honeycrisp’ apples. horttech. 2004;14(3):342–9. doi: 10.21273/horttech.14.3.0342

[32] Saure MC. Calcium translocation to fleshy fruit: its mechanism and endogenous control. Sci Hortic. 2005;105(1):65–89. doi: 10.1016/j.scienta.2004.10.003

[33] Carra B, Serra S, Knoche M, Rudell D, Musacchi S. Green spot in “WA 38”: growth dynamics, vascular integrity and architecture, and a hypothetical mechanism. PLoS One. 2026;21(7):e0351512. doi: 10.1371/journal.pone.0351512 42430424 PMC13354105

[34] Knoche M, Khanal BP, Brüggenwirth M, Thapa S. Patterns of microcracking in apple fruit skin reflect those of the cuticular ridges and of the epidermal cell walls. Planta. 2018;248(2):293–306. doi: 10.1007/s00425-018-2904-z 29705975

[35] Khanal BP, Grimm E, Finger S, Blume A, Knoche M. Intracuticular wax fixes and restricts strain in leaf and fruit cuticles. New Phytol. 2013;200(1):134–43. doi: 10.1111/nph.12355 23750808

[36] Straube J, Chen Y-H, Khanal BP, Shumbusho A, Zeisler-Diehl V, Suresh K, et al. Russeting in apple is initiated after exposure to moisture ends: molecular and biochemical evidence. Plants (Basel). 2020;10(1):65. doi: 10.3390/plants10010065 33396789 PMC7824318

[37] Knoche M, Peschel S. Deposition and strain of the cuticle of developing European plum fruit. J Amer Soc Hort Sci. 2007;132(5):597–602. doi: 10.21273/JASHS.132.5.597

[38] Brown K, Considine J. Physical aspects of fruit growth: stress distribution around lenticels. Plant Physiol. 1982;69(3):585–90. doi: 10.1104/pp.69.3.585 16662254 PMC426259

[39] Griffith C, Einhorn TC. The role of xylem in bitter pit incidence of apple: a review. J Am Pomol Soc. 2022;76(3):125–35.

