# Supplementary material for: Changes in skin properties are consequence but not cause of green spot in 'WA 38' apples
Source: PLoS One. 2026 Aug 19;21(8):e0356529. doi: 10.1371/journal.pone.0356529 (PMC13489476; doi:10.1371/journal.pone.0356529)
Supplement: Supplementary file 1 [file pone.0356529.s003.docx]

# Supporting information

**S1 Table. Mass of the cuticular membrane (CM) and strain release on excision and isolation of the CM in mature 'WA 38', 'Honeycrisp', and 'Enterprise'.**

| **Position** | **CM mass (g m^-2^)** | | | | **Strain release (%)** | | | |
| --- | --- | --- | --- | --- | --- | --- | --- | --- |
|  | **WA 38** | **Honeycrisp** | **Enterprise** | **Mean _Position_** | **WA 38** | **Honeycrisp** | **Enterprise** | **Mean _Position_** |
| **Calyx** | 20.0 ± 0.4 | 19.1 ± 0.6 | 22.2 ± 0.8 | 20.4 ± 0.4 | 7.1 ± 0.5 | 4.6 ± 0.4 | 5.3 ± 0.4 | 5.7 ± 0.3 b |
| **Calyx-equator** | 19.3 ± 0.4 | 18.7 ± 0.5 | 22.4 ± 0.8 | 20.2 ± 0.4 | 9.2 ± 0.5 | 5.9 ± 0.6 | 5.6 ± 0.4 | 6.8 ± 0.4 a |
| **Equator** | 19.7 ± 0.4 | 19.1 ± 0.5 | 22.8 ± 0.7 | 20.4 ± 0.4 | 9.2 ± 0.4 | 6.6 ± 0.5 | 4.8 ± 0.4 | 6.9 ± 0.4 a |
| **Equator-stem end** | 19.5 ± 0.3 | 18.8 ± 0.4 | 22.4 ± 0.5 | 20.2 ± 0.3 | 8.4 ± 0.5 | 6.6 ± 0.5 | 4.7 ± 0.5 | 6.6 ± 0.3 ab |
| **Stem end** | 19.9 ± 0.3 | 19.8 ± 0.3 | 22.4 ± 0.5 | 20.7 ± 0.3 | 7.8 ± 0.4 | 5.0 ± 0.4 | 4.1 ± 0.6 | 5.6 ± 0.3 b |
| **Mean _Cultivar_** | 19.7 ± 0.2 b | 19.1 ± 0.2 b | 22.4 ± 0.3 a |  | 8.3 ± 0.2 a | 5.7 ± 0.2 b | 4.9 ± 0.2 c |  |

Significant main effect for ‘position’ and CM mass, and strain release. Means followed by the same letter are not significantly different according to Tukey’s studentized range test (P=0.05).
